# Regulatory mechanisms of autophagy-related ncRNAs in bone metabolic diseases

**DOI:** 10.3389/fphar.2023.1178310

**Published:** 2023-12-07

**Authors:** Binghan Yan, Zhichao Li, Hui Su, Haipeng Xue, Daodi Qiu, Zhanwang Xu, Guoqing Tan

**Affiliations:** ^1^ College of First Clinical Medicine, Shandong University of Traditional Chinese Medicine, Jinan, China; ^2^ Affiliated Hospital of Shandong University of Traditional Chinese Medicine, Jinan, China

**Keywords:** autophagy, ncRNAs, bone metabolism, osteoporosis, osteoarthritis, rheumatoid arthritis

## Abstract

Bone metabolic diseases have been tormented and are plaguing people worldwide due to the lack of effective and thorough medical interventions and the poor understanding of their pathogenesis. Non-coding RNAs (ncRNAs) are heterogeneous transcripts that cannot encode the proteins but can affect the expressions of other genes. Autophagy is a fundamental mechanism for keeping cell viability, recycling cellular contents through the lysosomal pathway, and maintaining the homeostasis of the intracellular environment. There is growing evidence that ncRNAs, autophagy, and crosstalk between ncRNAs and autophagy play complex roles in progression of metabolic bone disease. This review investigated the complex mechanisms by which ncRNAs, mainly micro RNAs (miRNAs), long noncoding RNAs (lncRNAs), and circular RNAs (circRNAs), regulate autophagic pathway to assist in treating bone metabolism disorders. It aimed at identifying the autophagy role in bone metabolism disorders and understanding the role, potential, and challenges of crosstalk between ncRNAs and autophagy for bone metabolism disorders treatment.

## 1 Introduction

Bone is usually in a single constant state with standardized structure and function. The key to bone metabolic homeostasis is the dynamic balance between osteoblasts (OBs)-mediated bone formation and osteoclasts (OCs)-mediated bone resorption (Suzuki et al., 2020). OBs are thought to be derived from bone marrow mesenchymal stem cells (BMSCs) (Shu et al., 2021). While, OCs are mainly responsible for bone destruction and resorption and are multinucleated giant cells developing from the hematopoietic stem cells (HSCs) of the mononuclear macrophage lineage (Bernhardt et al., 2015). Imbalance between OBs-mediated bone formation and OCs-mediated bone resorption can cause an imbalance in bone metabolism, further inducing bone metabolic diseases such as osteoporosis (OP), osteoarthritis (OA), and rheumatoid arthritis (RA) (Chen et al., 2018; Zhao et al., 2020). As a typical bone metabolism disorders, OP is characterized by reduced bone mass, decreased bone quality, loosened bone density, degraded bone microarchitecture, and subsequently increased bone fragility, elevating the risk of fracture (He and Chen, 2022). OA is a prevalent chronic joint disease characterized by synovial inflammation, progressive degeneration of cartilage and associated extracellular matrix (ECM), bone redundancy formation, and subchondral osteosclerosis (Hu and Ecker, 2021). The specific causes of OA are elusive, including gender, obesity, and genetic factors in addition to aging (Niu et al., 2009). RA is a chronic inflammatory and autoimmune disease characterized by immune cell infiltration, synovial endoplasia, cartilage degeneration, and subchondral bone erosion (Huang et al., 2021). Currently, bone metabolic diseases are treated conservatively and the available drugs only focus on improving the symptoms. Furthermore, the epigenetic modification is accepted as the standard treatment to address the root cause, and it will be used as a biomarker and a protocol for diagnosis and treatment of related diseases. In addition, autophagy is crucial in bone metabolic diseases. Autophagy and epigenetics are emerging as potential stocks of new targets for bone metabolic diseases treatment and are becoming a hot topic for researchers.

Macro-autophagy/Autophagy is an intracellular degradation system that maintains dynamic cellular homeostasis by removing misfolded proteins and damaged organelles and macromolecules through lysosome-mediated autophagy and by providing necessary materials for cellular reconstruction, regeneration, and repair (Parzych and Klionsky, 2014). Due to the recycling properties and continuous bone remodeling, autophagy is a key regulator of bone metabolism (Duan et al., 2020).

NcRNAs, including micro RNAs (miRNAs/miRs), long noncoding RNAs (lncRNAs), and circular RNAs (circRNAs), importantly regulate the cellular processes such as cell proliferation, differentiation, apoptosis, and autophagy. An increasing number of studies have shown that bone metabolic processes are epigenetically regulated by miRNAs, lncRNAs, and circRNAs (Feng et al., 2018; Patil et al., 2020).

Both ncRNAs and autophagy are involved in the pathogenesis of bone metabolic diseases (Shen et al., 2016). Recent studies have shown that ncRNAs regulate autophagy through different molecular pathways, which in turn affect the progression of bone metabolic diseases. To date, there is no review focusing on the interrelationship among ncRNAs, autophagy, and bone metabolic diseases. Therefore, this paper briefly described the process of autophagy, focusing on the role of autophagy in different types of bone development and metabolic cells (including BMSCs, OBs, osteocytes, chondrocytes, and OCs). Based on recent studies, this paper reviewed the autophagy-related ncRNAs in bone metabolic diseases (including OP, OA, and RA). It aimed to offer a theoretical basis for exploring the pathogenesis and targeting the therapeutic potential of bone metabolic diseases.

## 2 Initiation and regulation of autophagy

**FIGURE 1:**
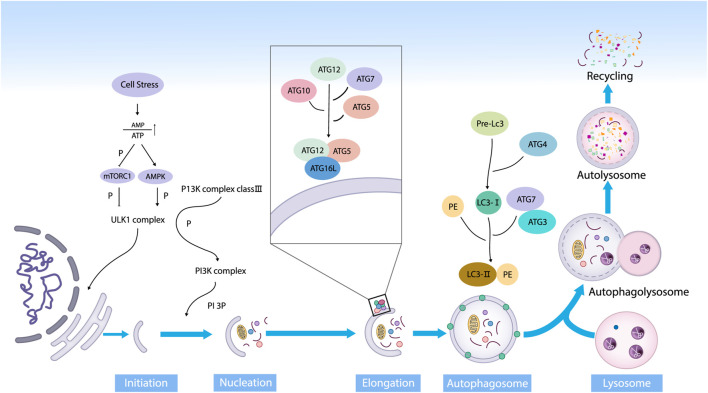
The main stages of autophagy. Autophagy is an evolutionarily conserved dynamic way in the form of degradation, and it mainly consists of initiation, nucleation, prolongation, maturation, and degradation (Abbreviations: ATP, adenosine triphosphate; AMP, adenosine monophosphate; mTOR, mammalian target of rapamycin; AMPK, 5ʹ AMP-activated protein kinase; ULK1, UNC-51-like kinase; PI3K, phosphoinositide3 kinase; PI3P, phosphatidylinositol 3-phosphate; Akt, protein kinase B; ATG, autophagy-related protein; LC3, microtubule-associated protein 1 light chain 3; PE, phosphatidylethanolamine).

Autophagy can be classified into three categories: chaperone-mediated autophagy, micro-autophagy, and macro-autophagy. Macro-autophagy is the main type and has been studied extensively (Mizushima and Komatsu, 2011). Macro-autophagy is referred to as autophagy in this paper. Autophagy can be induced by various factors inside and outside the body’s cells, including nutrient deficiencies, proteotoxic aggregates, proteotoxic aggregates, oxidative stress, deoxyribonucleic acid (DNA) damage, and microbial infections (Nakatogawa, 2020). According to the views of Galluzzi et al., autophagy can be divided into five stages (Galluzzi and Green, 2019). (1) In the initiation phase, nutrient deficiencies elevate the adenosine monophosphate (AMP)/adenosine triphosphate (ATP) ratios, which inhibits the mammalian target of rapamycin complex-1 (mTORC1) dephosphorylation inactivation. Followed that, the adenosine monophosphate-activated protein kinase (AMPK) is activated. Then, the UNC-51-like kinase1 (ULK1) complex (composed of ULK1, autophagy related 13 (ATG13), autophagy related 101 (ATG101), and family kinase-interacting protein of 200 kDa in a multiprotein complex) is activated, which is directly involved in the initiation of autophagic progenitor formation. (2) During the phagocytic vesicle nucleation, AMPK continues to activate ATG14, AMBRA1, UVRAG, Beclin-1 and other nucleation-related proteins, triggering the phagocytic nucleation when the PI3P is generated under the influence of supramolecular complexes with class III phosphoinositide-3 kinase (PI3K) activity. (3) In the prolongation phase, the autophagy-associated proteins ATG7 and ATG10 sequentially act on ATG12 to form a complex named ATG12 - ATG5 - ATG16L1. On the other hand, ATG4 cleaves the light chain 3 (LC3) progenitor protein into LC3-Ⅰ, which is then jointly cleaved by ATG7 and ATG3 into the mature form LC3-Ⅱ and bound to phosphatidylethanolamine (PE). The two sides bind and gradually extend with the support of WIPI protein until the substrate is wrapped, forming autophagic vesicles. (4) In the maturation stage, the autophagosome fuses with the lysosome and releases the substrate into the lysosome to produce autophagic lysosomes. (5) In the final degradation stage, acidic proteases acidify the lumen of the lysosomes and subsequently hydrolyze the autophagic lysosomes and their contents to remove out or reuse their hydrolyzed products such as amino acids, lipids, and nucleotides from the cells (Ravikumar et al., 2010) (Figure 1).

## 3 Autophagy and bone homeostasis

**FIGURE 2:**
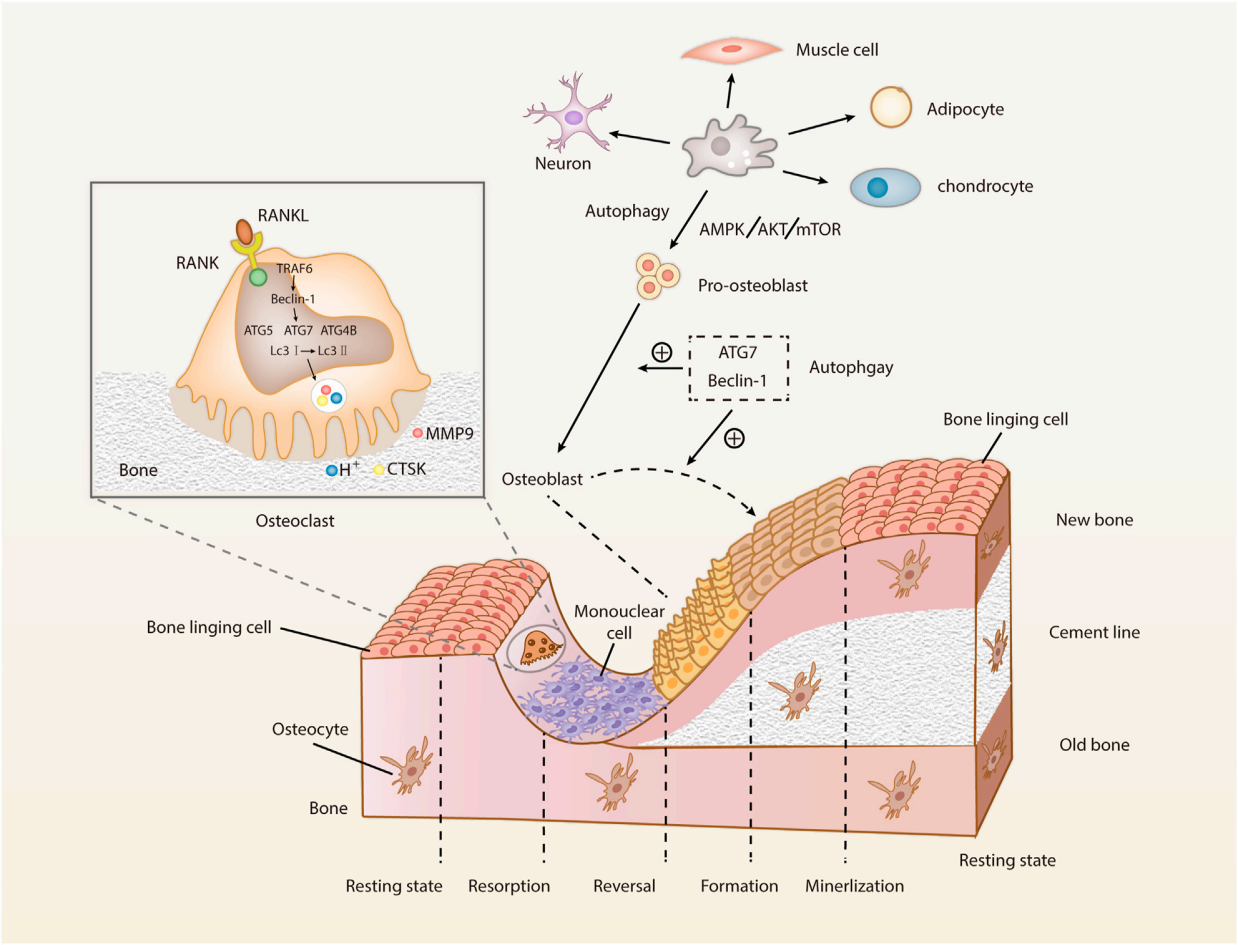
Cell types involved in bone metabolism and the roles of autophagy on OBs and OCs. Bone remodeling mainly refers to the OC-mediated bone resorption and OB-mediated bone formation. After the osteoblast differentiation, bone mineralization and bone reconstruction will be performed, marking the completion of bone remodeling. The dynamic balance between bone formation and degeneration is constantly harmonized (Abbreviations: RANK, receptor activator of NF-κB; RANKL, receptor activator of NF-κB ligand; TRAF6, tumor necrosis factor receptor-associated factor-6; Beclin-1, the autophagy markers; ATG, autophagy-related protein; LC3, microtubule-associated protein 1 light chain 3; CTSK, cathepsin K; MMM9, matrix metalloproteinase 9; mTOR, mammalian target of rapamycin; AMPK, 5ʹ AMP-activated protein kinase; Akt, protein kinase (B).

Bone is in a dynamic balance between bone formation and bone resorption, i.e., bone homeostasis, which is maintained by the tight cooperation of multiple types of cells, factors, hormones, and so on. OBs and chondrocytes derived from BMSCs build and shape the bone for maximum elasticity. OCs, which develop from HSCs of the mononuclear macrophage spectrum, maintain the dynamic balance of minerals by resorbing extensive surface of cancellous bone. Such balance between bone formation and resorption maintains a healthy development of bone (Zaidi, 2007). Osteocytes are derived from OB end-stage differentiation, highly active cells integral to normal skeletal function, surrounded by mineralized bone matrix, and regulate OBs and OCs by sensing the changes in stress and hormone levels (Tresguerres et al., 2020). Due to the cyclic nature of autophagy and the continuous bone remodeling, it is reasonable assumed that autophagy is relevant to the bone homeostasis and involved in the regulation of BMSCs, OBs, osteocytes, chondrocytes, and OCs (Figure 2).

### 3.1 Autophagy in BMSCs

Marrow mesenchymal stem cells (MSCs) have the ability of self-renewal and multipotential differentiation, and can proliferate and differentiate into a variety of cell types, such as OBs, chondrocytes, and adipocytes. As a type of MSCs, BMSCs present in the bone marrow and generate most osteogenic progenitor cells required for bone tissue development, bone metabolism, bone remodeling, bone repair, and bone regeneration. In terms of osteogenic differentiation of BMSCs, a large of undegraded autophagic vacuoles or autophagosomes can be accumulated in undifferentiated BMSCs (Nuschke et al., 2014). Meanwhile, lots of autophagosomes are accumulated within the cytosol of BMSCs during the early osteogenic differentiation, which may be driven by elevated bioenergetic demands. It indicates that autophagy is strongly related to the maintenance of stemness in BMSCs. In addition, BMSCs accumulate autophagosomes in the stem cell state and pass them to lysosomes at the beginning of differentiation, suggesting a close relationship between autophagy-related metabolism and BMSCs differentiation. Also, autophagy is involved in maintaining the stemness of MSCs derived from cord blood (Kim et al., 2020). A study showed that the AMPK pathway regulates the autophagy through early inhibition of mammalian target of rapamycin (mTOR) and Akt/mTOR, thereby regulating the osteogenic differentiation of MSCs (Pantovic et al., 2013). In addition, the balance of “ osteogenic-lipogenic” differentiation in BMSCs is integral to maintenance of bone homeostasis, and target Sirt3 can improv the autophagy and reversed the aging of BMSCs caused by advanced glycosylation end products (Guo et al., 2021). Whether the autophagy is in a basal level is a key factor to judge the survival of BMSCs after oxidative stress. Furthermore, Song et al. showed that the H_2_O_2_-mediated oxidative stress in BMSCs can cause massive cell death, and the increased intracellular autophagy can promote the survival of BMSCs at this time (Song et al., 2014a).

### 3.2 Autophagy in OBs

OBs are key components of the bone multicellular unit and exert a pioneering role in bone remodeling. Autophagy-related genes regulate autophagy and affect the activity and function of OBs. Specific knockdown of ATG7 triggers the endoplasmic reticulum stress (ERS) in OBs, reducing OBs and matrix mineralization (Li et al., 2018b). OBs are specialized mineralized cells. Apatite pinpoint-like structures in autophagic vesicles during mineralization suggest that autophagy is very important in implementing the physiological functions of OBs. The mineralization capacity of OBs is greatly reduced with the silencing of ATG7 and Beclin-1 by using the small interfering RNA (siRNA) technology (Nollet et al., 2014). The inhibition of autophagic flux hinders the outward delivery of minerals by OBs. Some other studies have confirmed that the mineralization of OBs can be stimulated by many substances by regulating autophagy, such as orthosilicic acid (Chi et al., 2019), VK2 (Li et al., 2019), and kaempferol (Kim et al., 2016), inducing cellular autophagy to stimulate differentiation and mineralization of OBs.

In addition, early initiation of autophagy can downregulate the oxidative stress within OBs and inhibit the apoptosis (Li et al., 2017). H_2_O_2_-induced oxidative stress damage in OBs can be mitigated by activation of the AMPK pathway that activates the autophagy to mitigate the corresponding damage (She et al., 2014). In addition to oxidative stress, the autophagy also can weaken the OBs apoptosis in other stressful environments, such as tumor necrosis factor-alpha (TNF-α) (Zheng et al., 2020), lipotoxicity (Al Saedi et al., 2020), and glucocorticoids (Wang et al., 2019). In addition, autophagy is involved in the osteogenic differentiation mediated by biological signals in the organism, for example, an insulin-like growth factor promotes osteogenesis by upregulating autophagy through the AMPK pathway (Xi et al., 2016).

However, some studies have shown that autophagy negatively regulate the OBs. Generation of reactive oxygen species (ROS) induced by dexamethasone upregulate the autophagy markers (such as LC3-II, Beclin-1, and p62) and autophagy, resulting in apoptosis in mouse embryonic OB progenitor cells (MC3T3-E1). Thus, the relationship between the degree of autophagic activity and OBs is complex, and autophagy has a bidirectional regulatory effect on OBs, the detailed mechanism of which remains to be investigated.

### 3.3 Autophagy in osteocytes

Located in mineralized bone matrix with low vascular supply, osteocytes are terminally differentiated cells generated from OBs. During the differentiation into bone cells, OBs undergo a drastic transformation in morphology, spatial location, and composition, which requires active recycling of organelles to provide nutrition and adapt to a hypoxic environment (Jaber et al., 2019). Furthermore, the long lifespan and relatively hypoxic and nutrient-deprived environment of osteocytes confirm that autophagy is crucial to maintain the osteocytes homeostasis and implement the physiological functions. Additionally, specific knockout of ATG7 mice exhibited an elevated oxidative stress in osteocytes and altered the bone remodeling with reduced bone mass and an aging phenotype of bone tissue (Onal et al., 2013).

The osteocyte network is a mechanosensory system of skeletal tissue, where osteocytes sense the mechanical loads and translate the resulting mechanical signals into various mechanical stimuli such as fluid shear stress (FSS), hydrostatic pressure, and direct deformation of osteocytes (Klein-Nulend et al., 2013). It has been found that cyclic mechanical stretch changes the osteocyte morphology and promotes the autophagy (Inaba et al., 2017), and FSS stimulates the induction of autophagy in osteocytes and accelerates ATP production and release (Zhang et al., 2018a).

### 3.4 Autophagy of chondrocytes

Autophagy is involved in cartilage formation. Deficiency of the ATG5 and ATG7 slows the chondrocyte proliferation and exacerbates the chondrocyte death (Vuppalapati et al., 2015). Targeted deletion of ATG5 in chondrocytes accelerates the production of ROS (López de Figueroa et al., 2015) and promotes the age-related OA (Bouderlique et al., 2016). The targeted deletion of ATG7 slows down the growth and shortens the bone length in mice (Horigome et al., 2020). Meanwhile, it can block the transportation of type II pre-collagen so that it is remained within the endoplasmic reticulum (ER), disrupting the integrity of ECM (Cinque et al., 2015). Thus, the autophagy protects chondrocytes from oxidative stress and preserves the functionality and integrity of ECM.

Formation and longitudinal growth of bone depend mainly on the endochondral osteogenesis, i.e., cartilage ossification, at the level of the cartilage growth plate which is composed of chondrocytes and ECM. The chondrocytes in the growth plate shows low regeneration rate and low vascularity, so that they are susceptible to hypoxia and nutrient deficiency, so it requires constant activation of autophagy by chondrocytes to maintain the normal cellular function. As a transcription factor, hypoxia-inducible factor-1α (HIF-1α) is sensitive to changes in oxygen levels and can allow chondrocytes to adapt to an avascular and hypoxic environment by regulating the autophagy, maintaining the chondrocyte viability (Lu et al., 2021b). Chondrocytes in a hypoxic environment rely on autophagosomes to selectively wrap the glycogen and generate the metabolic energy through anaerobic glycolysis, which are known as glycophagy and gluconeogenesis, respectively (Ueno and Komatsu, 2017). Such anaerobic glycolysis can be promoted by HIF-1α (Hollander and Zeng, 2019). In contrast, OB metabolizes glucose to lactic acid even in the presence of oxygen (Guntur et al., 2014), i.e., aerobic glycolysis. Various types of osteocytes maintain their physiological roles through specific metabolic pathways, and maintaining the homeostasis among skeletal cells is closely related to the proper way of energy metabolism.

### 3.5 Autophagy in OCs

OCs are highly differentiated multinucleated giant cells developed from HSCs of the mononuclear macrophage lineage and fused with the mononuclear progenitor cells. Meanwhile, OC is an irreplaceable component of the immune system of the body due to the presence of the macrophage-OC axis and nuclear factor-κB (NF-κB) and nuclear factor of activated T-cell c1 (NFATc1) signaling in OCs formation. In addition to this, OCs dominate the bone resorption. With the macrophage colony-stimulating factor (M-CSF), HSCs convert the tissue-specific macrophage colony-forming units (CFU-M), which are the progenitor cells of OCs. Furthermore, the CFU-M will be differentiated into multiple mononuclear OCs when they are activated by receptor activator of NF-κB ligand (RANKL) - receptor activator of NF-κB (RANK) signaling. These mononuclear OCs will fuse to form multinucleated OCs, which then attach to the bone resorption area and become mature OCs, secreting acid (H^+^), cathepsin K (CTSK), and matrix metalloproteinase 9 (MMP9). The cells are then polarized, resulting in a closed area, fold margin, which is attached to the bone surface, forming many closed compartments around it. The bone minerals are dissolved firstly, followed by the degradation of bone matrix (collagen, non-collagenous proteins, etc.). The degraded bone matrix enters the OCs through the border of the folds by endocytosis and is excreted by OCs after phagocytosis and transport. Finally, OCs are detached from the bone surface and transferred to new sites.

Autophagy promotes the differentiation of OCs progenitors into OCs. The RANKL/RANK/OPG system mediates the differentiation of OCs, with Ca^2+^/calcineurin/NFATc1 being a major signaling pathway in this system (Zhang et al., 2017). This signaling pathway is regulated by Ca^2+^ oscillations and inhibits the differentiation of OCs by inhibiting autophagy through transient receptor potential vanilloid 4 (TRPV4) Ca^2+^ channel and P2X7 receptor (P2X7R) Ca^2+^ channel (Cao et al., 2019; Ma et al., 2022). Multiple autophagy-related proteins (including ATG5, ATG7, ATG4B, and LC3) and Beclin-1 are involved in the differentiation of OCs, and the expressions of ATG5, ATG7, ATG4B, and Beclin-1 are increased in RANKL-stimulated macrophages. RANKL-mediated ATG activation and differentiation of OCs decreased when Beclin-1 is inhibited. Meanwhile, upon simulation by RANKL, the tumor necrosis factor receptor-associated factor-6 (TRAF6), in a RANKL dependent manner, subsequently initiates ATG and subsequent differentiation of OCs by mediating Beclin-1 ubiquitination (Arai et al., 2019). Furthermore, RANKL induces autophagy in OC progenitor cells and promotes generation of OCs by positively affect the Bcl-2 phosphorylation at the Ser70 locus, suggesting that RANKL promotes the generation of OCs through the Bcl-2/Beclin-1 pathway (Ke et al., 2022). Nepetin, a natural active ingredient, has been shown to block TRAF6-mediated Beclin-1 ubiquitination, inhibit autophagy and progenitor cell fusion, and reduce bone resorption of OCs. Studies have shown that ATG4B, ATG5, and ATG7 promote the functions of OCs, and their deficiencies inhibit the conversion of LC3-I to LC3-II and the maturation of autophagosomes, thus affecting the bone resorption of OCs (DeSelm et al., 2011; Yu et al., 2019b; Hiura et al., 2022). Epigenetically, the autophagy related pathways of methyltransferase-like 14 (METTL14)/Beclin-1 and Tet methylcytosine dioxygenase 2 (TET2)/Beclin-1 are involved in the formation of OCs genesis, with the former inhibiting the differentiation of OCs (He et al., 2022) and the latter promoting the differentiation of OCs (Yang et al., 2022). The migration and bone resorption ability of OCs are mainly determined by their adaptive morphology and plantar formation and degradation. LC3-II is required for pedicle turnover and OC migration. Insufficient LC3-II cannot degrade the Kindlin3, disassembling the inability of the discarded pedicle, increasing the pedicle and disorder of intracellular actin structure, and impairing the migration of OCs, migration of which on the bone surface is crucial for the bone resorption function (Zhang et al., 2020).

## 4 ncRNAs: the link between epigenetics and genetics

Epigenetic abnormalities can result in malignancies, immune diseases, and metabolic diseases (Letarouilly et al., 2019; Yang et al., 2020d; Pang et al., 2021). Recent studies have shown that epigenetic abnormality is an important cause of bone metabolic diseases (Li et al., 2020c). Epigenetics generally refers to heritable phenotypic changes that do not involve the changes in nucleotide sequences and are caused by DNA methylation, histone modifications, chromatin remodeling, and the regulation of ncRNAs.

The roles of ncRNAs in regulating the epigenetics of bone metabolic diseases have attracted extensive attention (Husain and Jeffries, 2017; Raje and Ashma, 2019; Chan et al., 2022). miRNAs are small ncRNAs of approximately 21–22 nucleotides (nt) length, originating from the self-folding region of RNA transcripts. They are the most studied ncRNAs that cleaves 3′ -untranslated region (3′-UTR) target mRNAs in a base-pairing manner and promotes specific mRNAs degradation and/or translation inhibition at the post-transcriptional level, playing a key role in biological processes as a novel gene expression regulator (Bartel, 2004; Behm-Ansmant et al., 2006). Catalyzed by RNA polymerase II (pol II), the genes of these miRNAs generate primary miRNAs (pri-miRNAs), which are thousands of bases long, have a 5′ cap and a 3′ tails, along with one or more localized hairpin-like structures, and exist in polycistronic form (Okamura et al., 2013). The cleavage and processing from pri-miRNAs to pre-miRNAs require a role in the nucleus called Microprocessor Complex, which consists of the ribonuclease DROSHA and DGCR8 in the nucleus (Denli et al., 2004; Gregory et al., 2004; Han et al., 2004). The resulting pre-miRNAs are transported to the cytoplasm via Exportin-5. Here, it is sheared into a shorter double-stranded miRNA precursor (miRNA duplex) by the RNase-III enzyme (Dicer) and its co-factor. Subsequently, the two strands of the miRNA duplex are separated by RISC. with one evolving into a mature miRNA, which enhances the degradation and/or translational repression of specific mRNAs at the post-transcriptional level. (Chen et al., 2019). Any small changes or epigenetic modifications in the gene processing of miRNAs during the above processes can cause defected expression of miRNAs (Farooqi et al., 2019). Numerous studies have shown that disorders of miRNAs are tightly related to the formation and progression of bone metabolic diseases (Ding et al., 2021).

The lncRNAs are ncRNAs over 200 nt in length, and most of them are transcribed by RNA polymerase II. Based on the location of their parent genes in the genome, lncRNAs can be classified into five categories: righteous lncRNAs, antisense lncRNAs, intronic lncRNAs, bidirectional lncRNAs, and long-stranded intergenic ncRNAs (Ulitsky and Bartel, 2013; Fang and Fullwood, 2016). lncRNAs possess the biological functions as signaling molecules, molecular inducers, guidance molecules, and molecular scaffolds. In particular, they can act as miRNAs “sponges” and regulate the activity of mRNAs through competitive adsorption of miRNAs (Bridges et al., 2021). In addition, they regulate the expression of specific genes at the transcriptional, translational, and epigenetic levels (Xing et al., 2021).

**FIGURE 3:**
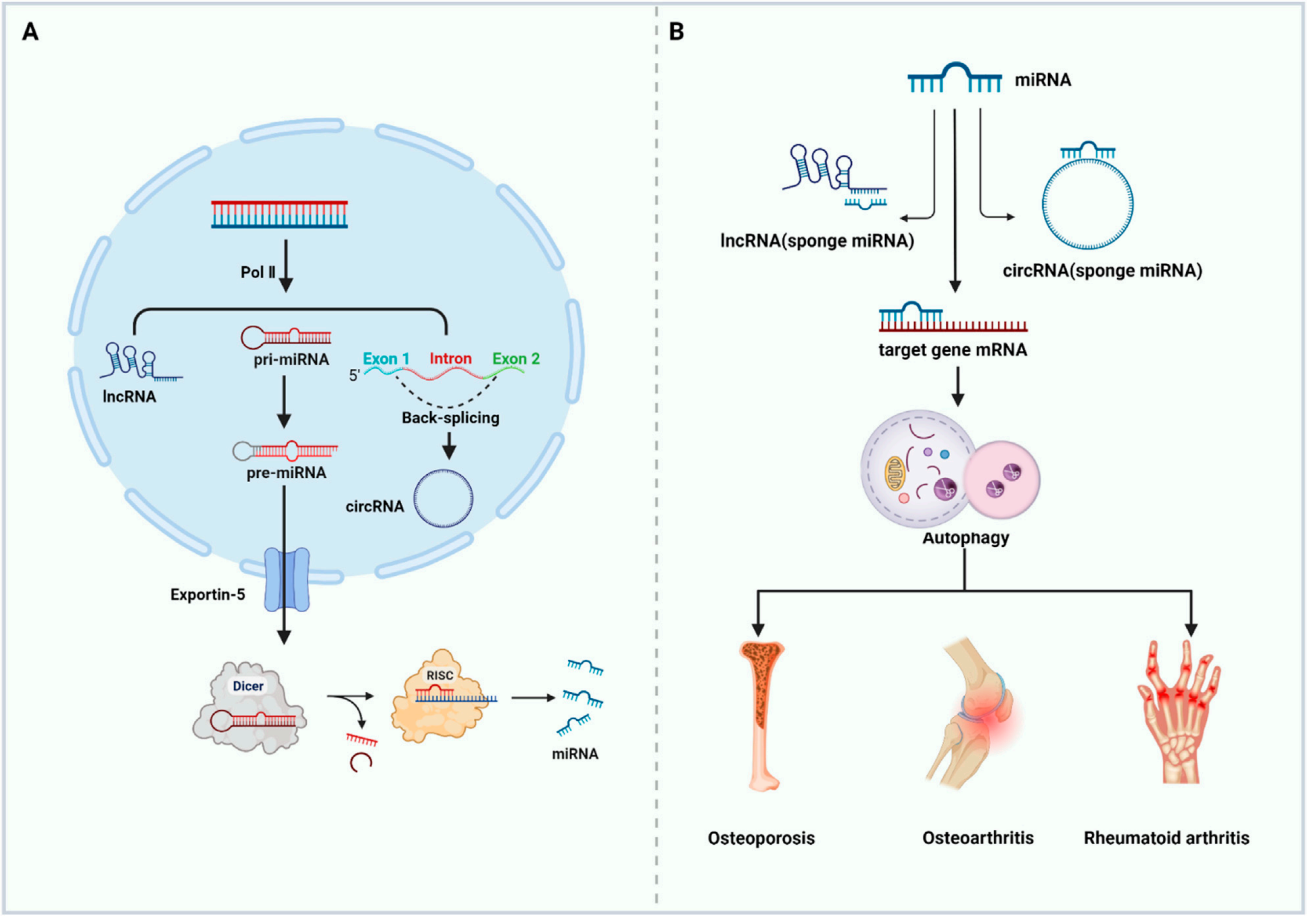
ncRNAs regulate autophagy and participate in the occurrence and development of bone metabolic diseases. **(A)** Biogenesis of ncRNAs **(B)** ncRNAs target autophagy related genes to regulate autophagy and then affect the occurrence and development of bone metabolic diseases (Abbreviations: Pol II, RNA polymerase II; pri-miRNA, primary miRNA; pre-miRNA, the precursor miRNA; RISC, RNA-induced silencing complex). Created with https://www.BioRender.com.

CircRNAs are transcribed by Pol II and circularized through back-splicing. CircRNAs are highly conserved, single-stranded circular endogenous ncRNAs with a length range of several hundred to several thousand nucleotides and lacking free 5′ end caps or 3′ tails. circRNAs exhibit a better miRNAs affinity than other competing endogenous RNAs (ceRNAs), hence they are also known as “super sponges” (Jeck et al., 2013; Tay et al., 2014; Afzali and Salimi, 2019). Due to the closed-loop structure, circRNAs are resistant to RNA exonucleases and highly stable and are therefore considered as ideal biomarkers for disease diagnosis and prognosis (Zhang et al., 2018b) (Figure 3).

## 5 Autophagy-relate ncRNAs and bone metabolic diseases

Occurrence and progression of bone metabolic diseases are greatly determined by bone homeostasis. Mechanical stimuli, epigenetic regulation, and hormonal factors can cause the imbalance of bone homeostasis and disorders of bone metabolism. At the cellular level, this is often manifested by abnormalities in the morphology and function of BMSCs, OB, OC, chondrocytes, and bone cells. ncRNAs, as important regulators, exert critical regulatory roles in cellular processes such as cell proliferation, differentiation, apoptosis, and autophagy, of which autophagy plays an important role in circulating the cellular components and maintaining bone homeostasis, as mentioned above. The autophagy-related ncRNAs have been demonstrated to play a novel and critical role in bone metabolic diseases. Impacts of crosstalk between ncRNAs and autophagy on bone homeostasis should to be fully analyzed for a better understand on the pathogenesis and clinical therapeutic potential of the interaction between them.

### 5.1 Autophagy-relate ncRNAs and OP

OP is a chronic metabolic bone disease characterized by loosened bone density, weaker bone tissue microarchitecture, increased bone fragility, and an increased risk of fracture. It is widely accepted that bone remodeling is the primary process in adult skeleton under normal conditions. During this process, the amount of bone resorption equals to that of bone formation in each subsequent bone remodeling unit (the bone remodeling equilibrium) (Eastell and Szulc, 2017). If such balance cannot be maintained, OP will occur. OP can be primary or secondary. The primary OP includes postmenopausal OP and senile OP. Postmenopausal estrogen deficiency will increase the osteoclastic bone resorption, which is the main pathogenesis of postmenopausal OP, and autophagy mainly regulates the osteoclastic bone resorption at this stage (Li et al, 2020b). For a senile OP, autophagy levels decrease with cellular senescence, resulting in bone loss, and autophagy defects in BMSCs lead to an imbalance in osteogenic and lipogenic differentiation.

As mentioned above, ncRNAs include miRNAs, lncRNAs, and circRNAs, which play important regulatory roles in cellular autophagy. 70 lncRNAs, 260 circRNAs, and 13 miRNAs were differentially expressed between OP patients and normal subjects, which is determined using the Illumina deep sequencing technology by Jin et al. (2018). These differences imply that ncRNAs can serve as potential disease biomarkers and therapeutic targets for treating OP. Furthermore, there are complex interactions between ncRNAs and the autophagy process. They directly or indirectly regulate autophagy-related factors or signaling pathways, thereby controlling the osteogenic differentiation of BMSCs, the proliferation and differentiation of OBs, and the differentiation of OCs, which in turn influence the occurrence and development of OP.

#### 5.1.1 Autophagy-relate miRNAs regulate osteogenic differentiation of BMSCs

Osteogenic differentiation of BMSCs is crucial in maintaining bone homeostasis, providing a cellular source for bone growth and repair. Recent studies have identified several miRNAs associated with the osteogenic differentiation of BMSCs and their regulatory relationship with autophagy. These findings provide new insights into the understanding and treatment of bone diseases. MiRNAs can regulate autophagy levels by modulating autophagy-related signaling pathways or proteins, thereby controlling the osteogenic differentiation of BMSCs. miR-223-3p overexpression inhibits the autophagy by targeting Forkhead box O3 (FOXO3), thus suppressing the osteogenic differentiation of BMSCs (Long et al., 2021). Meanwhile, miR-140-3p promotes the autophagy by targeting the sprouty-related EVH1 domain-2 (Spred2), thereby regulating the osteogenic differentiation of BMSCs (Liu et al., 2021). Besides, miR-152-5p inhibited the ATG14-mediated autophagy, thereby inhibiting the osteogenic differentiation of BMSCs (Li et al., 2022). This finding suggests that miR-152-5p plays a negative regulatory role in the osteogenic differentiation of BMSCs by regulating autophagy. While both miRNAs and autophagy can regulate the osteogenic differentiation of BMSCs, there is limited research on how miRNAs specifically modulate BMSCs’ osteogenic differentiation by mediating autophagy-related factors. Further investigation into how miRNAs regulate BMSCs’ osteogenic differentiation by modulating autophagy-related factors will contribute to a better understanding of the mechanisms underlying bone metabolic disorders and provide novel therapeutic targets for relevant diseases. In addition, some autophagy-related ncRNAs can promote the differentiation of BMSCs in other directions. For example, Zhang et al. found that overexpressed miR-9 upregulated the amount of autophagy marker LC3 and LC3II/LC3Ⅰ ratio and upregulated the neuronal markers Nestin and microtubule-associated protein 2 (MAP2) (Zhang et al., 2015b). It suggests that overexpression of miR-9 promotes autophagy, enabling the MBSCs differentiate into neuronal cells and reducing the osteogenic differentiation. This “special” differentiation of BMSCs can also be regulated by ginseng-derived exosomes (G-Exos) through the incorporation of miRNAs (Xu et al., 2021).

#### 5.1.2 Autophagy-related miRNAs regulate the proliferation and differentiation of OBs

The dysregulation of the number or activity of OBs is closely related to the pathophysiological mechanisms of bone diseases such as OP. Activation of autophagy can enhance OBs differentiation and restore bone mass; moreover, miRNAs have been demonstrated to modulate bone formation, with their expression levels potentially exhibiting significant variations under healthy and pathological conditions, thereby serving as biological markers for OP (Valenti MT et al., 2018). Lu et al. found, by *in vivo* and *in vitro* experiments, that miR-15b was highly expressed in mice with ovariectomy, while ubiquitin specific peptidase 7 (USP7) and lysine (K)-specific demethylase 6B (KDM6B) were lowly expressed (Lu et al., 2021c). It confirms that inhibition of USP7 through overexpression of miR-15b further suppressed the expression of KDM6B and inhibited the proliferation, differentiation, and autophagy of OBs, hereby exacerbating OP. Their findings offer insights into a plausible pathophysiological mechanism, elucidating the association between OB dysregulation and bone diseases such as OP. Overexpression of miR-15b emerges as a potential therapeutic target; by manipulating the expression of USP7 and KDM6B, it is conceivable to influence OB functionality, hence ameliorating the pathophysiological processes of bone diseases. Nevertheless, further research is required to delve deeper into this mechanism and identify more effective treatment strategies. It is apparent that miRNAs can regulate autophagy levels by targeting relevant proteins; likewise, autophagy can maintain miRNA homeostasis by selectively degrading key components of miRNA biosynthesis (Shen et al., 2016). The cross-talk between miRNAs and autophagy constitutes a pivotal domain in the regulation of OBs and, by extension, osteoporosis; however, it is noteworthy that current research in this area is limited and the specific mechanisms remain unclear, warranting thoughtful consideration and in-depth exploration in future studies.

#### 5.1.3 Autophagy-relate miRNAs regulate the differentiation of OCs

OCs can realize bone resorption, and abnormalities in their functional activity are likely to cause OP. Transforming growth factor β1-activated kinase 1 binding protein 2 (TAB2) is a novel Beclin-1 interacting molecule and a co-activator of transforming growth factor-β activated kinase-1 (TAK1) in response to autophagy stimulation (Zhou et al., 2020a). Sul et al. found that the expression of miR-155 was significantly increased in OCs induced by lipopolysaccharide (LPS), and the targeting of miR-155 overexpression decreased the expression of TAB2, which dissociated Beclin-1 from the complex of TAB2 and Beclin-1 and promoted the binding of TAK1 to the complex. Meanwhile, the autophagy is formed by increasing the level of LC3-Ⅱ and decreasing the level of p62, thereby affecting the autophagy and increasing the formation and activity of OCs. The results confirmed the new roles of miR-155 in LPS-induced OCs in regulating autophagy by targeting TAB2 (Sul et al., 2018). Inhibiting the expression of miR-155 could serve as a therapeutic target to hinder the differentiation of OCs in an inflammatory environment by suppressing autophagy. In a hypoxic environment, the cells can be stressed to autophagy. Su et al. found that miR-20a was inhibited in a hypoxic environment, allowing the expression of autophagy markers (LC3-II and ATG16L1) and OCs differentiation markers (TRAF6, NFACT1, tartrate-resistant acid phosphatase, etc.) (Sun et al., 2015). When the miR-20a is overexpressed, targeting ATG16L1 inhibits the autophagy and downregulates the expression of markers of autophagy and differentiation of OCs. It indicates that miR-20a can act as a target to inhibit the differentiation of OCs in hypoxic environments by suppressing autophagy. Autophagy is one of the cellular survival mechanisms under various stress conditions, necessitating stringent regulation to aptly respond to diverse stimuli, thereby adapting to a constantly changing environment. Different stress scenarios can instigate autophagy, possibly accompanied by the suppression or expression of certain miRNAs. Researchers seek to explore the impact on OCs by analyzing the interactions between autophagy and miRNAs. In summary, it is evident that miRNAs can regulate OCs autophagy by modulating target genes, thereby influencing their differentiation and proliferation. Furthermore, a subset of miRNAs has been proven capable of affecting OCs quantity and even altering their volume. Franceschetti et al. verified the functions of highly upregulated miR-365 and miR-99b in OCs, and found that inhibiting the miR-365 increased the number of OCs but reduced their size. However, inhibiting the miR-99b reduced the volume and the number of OCs. Furthermore, they predicted, through the KEGG pathway analysis, that these two miRNAs regulated the mTOR and PI3K/Akt pathways (Franceschetti et al., 2014), but whether they were involved in cellular autophagy needs to be further verified.

#### 5.1.4 Autophagy-relate lncRNAs and circRNAs regulate OP-associated cells

Most of the lncRNAs and circRNAs act as sponges of miRNAs to influence the progression of OP. However, compared to extensively studied miRNAs, it is a relatively new but highly intriguing research direction. Zine finger antisense 1 (ZFAS1), a lncRNA, inhibits the autophagy and osteogenic differentiation of BMSCs by sponging miR-499, suggesting that ZFAS1 could be a new target for OP treatment (Wu et al., 2022). circRNA-0026827 can target spongy miR-188-3p to upregulate the Beclin-1-mediated autophagy, promoting the osteogenic differentiation of dental pulp stem cells (DPSCs) (Ji et al., 2020). Zhao et al. found that NEAT1 (a lncRNA) regulated the expression of hexokinase 2 (HK2) by sponging miR-144F-3P, and promoted the autophagy of OBs by regulating the miR-144F-3P/HK2 axis (Zhao et al., 2022). In addition, Dai et al. used LPS-stimulated human osteoblast-like cell (MG63) cells to construct a model of inflammation-stimulated OBs, and found 427 differentially expressed genes in LPS-stimulated MG63 cells. Among them, NEAT1 was significantly downregulated, LPS inflammatory cytokines and NLRP3 were upregulated, while the autophagy-related and osteogenesis-related proteins were inhibited, which altered the cell cycle and promote apoptosis. However, this effect was reversed by overexpression of NEAT1, confirming that LPS-induced inflammation in MG63 cells can be ameliorated by NEAT1 through activation of autophagy and inhibition of NLRP3 inflammatory vesicles (Dai et al., 2021). In addition, overexpression of TUG 1 (a lncRNA) increases the LC3II/Ⅰ ratio and the number of autophagosomes, which promotes the osteogenic differentiation of BMSCs. When the TUG 1 is overexpressed, AMPK and p53 are elevated, and level of mTOR is decreased, suggesting that TUG 1 can regulate the AMPK/mTOR/autophagy axis to promote the osteogenic differentiation of BMSCs (Lu et al., 2021a). Although the research on lncRNAs and circRNAs is currently limited, existing studies suggest that both have potential roles in regulating autophagy and osteoporosis. Current research mainly focuses on the osteogenic differentiation of BMSCs and the proliferation and apoptosis of osteoblasts. However, there is limited exploration of osteoclasts and other aspects. This undoubtedly presents a promising new direction for future research. Table 1 lists the autophagy-related ncRNAs for OP treatment.

**TABLE 1:** ncRNAs for OP treatment by targeting autophagy.

ncRNA	Inhibition/activati on of autophagy	Targets	Mechanism	References
miR-223-3p	Inhibition	FOXO3	Inhibiting the osteogenic differentiation of BMSCs	Long et al. (2021)
miR-140-3p	Activation	SPRED2	Promoting the osteogenic differentiation of BMSCs	Liu et al. (2021)
miR-152-5p	Inhibition	ATG14	Inhibiting the osteogenic differentiation of BMSCs	Li et al. (2022)
miR-9	Activation	LC3	Promoting the differentiation of BMSCs into neuronal cells	Zhang et al. (2015b)
miR-Let-7F	Inhibition	Beclin-1, ATG12, ATG5, and LC3	Promoting the osteogenic differentiation of BMSCs	Shen et al. (2022)
miR-15b	Inhibition	USP7 and KDM6B	Inhibiting the proliferation and differentiation of OBs	Lu et al. (2021c)
miR-155	Activation	TAB2	Promoting the differentiation of OCs in an inflammatory environment	Sul et al. (2018)
miR-20a	Inhibition	ATG16L1	Inhibiting the differentiation of OCs in a hypoxic environment	Sun et al. (2015)
lncR-ZFAS1	Inhibition	miR-499	Inhibiting the osteogenic differentiation of BMSCs	Wu et al. (2022)
lncR-NEAT1	Activation	miR-144F-3P/HK2	Inhibiting the formation of OBs	Zhao et al. (2022)
lncR-NEAT1	Activation	NLRP3	Inhibiting the apoptosis of OBs	Dai et al. (2021)
lncR-TUG1	Activation	AMPK/mTOR	Promoting the osteogenic differentiation of BMSCs	Lu et al. (2021a)
circR-0026827	Activation	miR-188-3P	Promoting the osteogenic differentiation of DPSCs	Ji et al. (2020)

Abbreviations: BMSCs, bone marrow mesenchymal stem cells; OBs, osteoblasts; OCs, osteoclasts; FOXO3, Forkhead box O3; Spred2, sprouty-related EVH1 domain-2; ATG, autophagy-related protein; LC3, microtubule-associated protein 1 light chain 3; Beclin-1, the autophagy markers; USP7, ubiquitin specific peptidase 7; KDM6B, lysine (K)-specific demethylase 6B; TAB2, transforming growth factor β1-activated kinase 1 binding protein 2; HK2, hexokinase 2; NLRP3, NOD-like receptor family pyrin domain containing 3; AMPK, 5ʹ AMP-activated protein kinase; mTOR, mammalian target of rapamycin.

### 5.2 Autophagy-relate ncRNAs and OA

OA is a common chronic degenerative joint disease characterized by cartilage degeneration, bone remodeling, bone redundancy, and synovitis (Millerand et al., 2019). Its pathogenesis is complex and unclear yet. Its pathological changes revolve around apoptosis and dysfunction of chondrocytes (Duan et al., 2020). Autophagy itself can induce cell death, or act in conjunction with apoptosis, or as a backup mechanism for cell death in the presence of defective apoptosis. In many cases, autophagy is initiated as a cytoprotective mechanism that inhibits the cell death, but there are also some cases which reveal that stimuli can induce both apoptosis and autophagy. There is increasing evidence that both ncRNA metabolism and autophagy are involved in generation and growth of OA. In addition to targeting and regulating the autophagy-related gene expression, ncRNAs can regulate the mTOR pathway and other key proteins of autophagy, including sirtuins 1(SIRT1) and transcription factors, such as FOXOs, to involve in the regulation of autophagy.

#### 5.2.1 ncRNAs regulate autophagy-related gene expression to regulate chondrocyte autophagy

ncRNAs can involve in the autophagy regulatory network and mediate the transcriptional regulation of autophagy-related genes. lncRNA-CIR exhibits a higher expression in OA chondrocytes than in normal cartilage, elevating the LC3-II and Beclin-1, which are key genes of autophagy, thus increasing the autophagy in chondrocytes and accelerated the degradation of the ECM (Wang et al, 2018a). Similarly, by targeted inhibition of ATG12 gene expression, LC3-II conversion, and autophagosome formation, miR-128 initiates the apoptotic program, reduces the chondrocyte proliferative capacity, and exacerbating the progression of OA (Sun et al., 2022). miRNA Let-7E directly regulates the autophagy-related genes, exhibits a low expression in the peripheral blood of OA patients, and is a promising candidate for predicting OA risk, because it is independent of age, sex, and body mass index (BMI) of OA patients. The lowly expressed Let-7E downregulates the Beclin-1 and LC3II/I and promotes apoptosis through inhibiting the autophagy. However, this “toxic effect” on chondrocytes can be alleviated by the Simiao San (a Chinese herbal soup), which regulated the Let-7E to normal expression and reversed apoptosis, thus acting against OA (Feng et al., 2020). Unlike Let-7E, circRAN-0037658 showed a high expression in OA chondrocytes. Sui et al. found that knockdown of circRAN-0037658 upregulated the LC3, downregulate the ATG5 and Beclin-1, and reduced the inflammatory factors such as TNF-α in chondrocytes (Sui et al., 2021). It suggests that knockdown of 0037658 could slow down the progression of OA.

#### 5.2.2 ncRNAs regulate chondrocyte autophagy via the mTOR pathway

mTORC1 is an important and negative regulator of autophagy. Previous evidence suggests that inhibiting the mTORC1 signaling downward may be a valuable strategy for OA prevention and treatment (Yang et al., 2020b). ncRNAs, especially miRNAs, can regulate autophagy through the mTOR pathway to affect the chondrocyte apoptosis, proliferation, and the degradation of ECM. Yu et al. found that highly expressed miR-206 could inhibit the chondrocyte autophagy and apoptosis by activating the insulin-like growth factor-1 -mediated PI3K/Akt/mTOR signaling pathway (Yu et al., 2019a). In contrast, miR-27a is directly targeted to silence the 3′-UTR of the PI3K gene, downregulates PI3K, and promotes OA chondrocyte autophagy and apoptosis by inhibiting the PI3K/Akt/mTOR pathway (Cai et al., 2019). miR-20 inhibits the chondrocyte autophagy and proliferation by directly targeting and regulating the ATG10 via the PI3K/Akt/mTOR pathway rather than inhibiting apoptosis (He and Cheng, 2018), suggesting that knockdown of miR-20 may be a means to combat OA. Upregulated circR-7 or downregulated miR-7 could activate the PI3K/Akt/mTOR pathway, inhibit the autophagy, and exacerbate the OA cartilage ECM degradation (Zhou et al., 2020b). Under normal circumstances, miR-155 can inhibits the autophagy by suppressing the ULK1, FOXO3, ATG14, ATG5, ATG3, gamma-aminobutyric acid receptor-associated protein-like 1 (GABARAPL1), and microtubule-associated proteins LC3. Although miR-155 can target rapamycin-independent component of mammalian target of rapamycin (a key component of mTORC2 that induces TORC-1 activation via Akt) to activate mTOR activity, its inhibitory mechanism for autophagy has little to do with mTOR activity (D'Adamo et al., 2016). However, when exposed to organic pollutants polychlorinated biphenyls (PCBs), miR-155 becomes more, which inhibited autophagy by activating PI3K/Akt/mTOR and Rictor/Akt/mTOR signaling pathways, promoting the progression of OA. At this point, the mechanism of miR-155 inhibiting autophagy was related to the activity of mTOR pathway activity (Liu et al., 2022). In the normal environment without pollutants, miR-31-5P is opposite to miR-155, which promotes autophagy and is inextricably linked to the activity of mTOR. In this way, it inactivates the mTORC1 signaling pathway by directly targeting SOX4, promoting autophagy and alleviating apoptosis in OA chondrocytes (Xu et al., 2022). To our knowledge, the TRAF protein transcribed by TNF-receptor-associated factor 3 (TRAF3) can activate NF-κB and cell death (Häcker et al., 2006). miR-107 inhibits the activation of Akt/mTOR and NF-κB pathways by targeting TRAF3, promoting autophagy and inhibiting apoptosis (Zhao et al., 2019). In addition, leptin elevates the expression of miR-LOXL3 and inhibits the chondrocyte autophagy, which may also be related to mTOR activation (Wei, 2022). In addition to miRs, lcnRNAs are involved in regulating the mTOR pathway. For example, lncRNA GAS5 is highly expressed in OA chondrocytes, increases with the progression of OA, and regulates mTOR to inhibit autophagy and promote apoptosis in OA chondrocytes by targeting miR-144 (Ji et al., 2021). GAS5, in addition to regulating the mTOR pathway, can directly sponge the miRs and affect the cells. Meanwhile, GAS5 can inhibit the effect of miR-21 on cell survival properties, and the overexpression of GAS5 increases the apoptosis and suppresses the autophagic response (Song et al., 2014b). Exosome ncRNAs are important exosome cargoes and have received widespread attention because of their extensive regulatory role in gene expression (Zhang et al., 2015c). Wen et al. focused on the lncRNA KLF3-AS1 mediated by exosomes derived from mesenchymal stem cells and found that KLF3-AS1 expression was upregulated in the damaged chondrocytes, which enhanced the chondrocyte viability and inhibited the apoptosis by activating the PI3K/Akt/mTOR signaling pathway, thus inhibiting autophagy (Wen et al., 2022).

#### 5.2.3 ncRNAs regulate chondrocyte autophagy through the autophagy key protein SIRT1 and the transcription factor FOXO3

SIRT1 regulates the autophagy at different steps from initiation to degradation, while its level and function are also regulated by the autophagy. SIRT1 directly activates autophagy in human chondrocytes to promote the chondrogenesis and prevent OA. Salvianolic acid B, one of the effective active ingredients in the Chinese medicine Salvia miltiorrhiza, can upregulate the lncRNA KCNQ1OT1, which acts as a sponge for miR-128-3p to elevate SIRT1 through the ceRNA network. Furthermore, salvianolic acid B activates the chondrocyte autophagy and reduces the chondrocyte apoptosis through the KCNQ1OT1/miR-128-3p/SIRT1 signaling pathway (Sun et al., 2022), which is another study on using the main active component of a natural product to prevent OA at the level of autophagy and epigenetic modifications. Natural products are a “treasure trove” that may have unexpected effects on OA prevention in the future. In addition, lncRNA POU3F3 can protect the chondrocytes by targeting the downregulation of FOXO3 through uptake of miR-29a-3p, thereby inhibiting the chondrocyte autophagy, enhancing cell activity, and slowing down the apoptosis (Shi et al., 2022).

In addition, ncRNAs can regulate the relationship between autophagy and chondrocytes by regulating other related proteins. RHOT1, a circRNA, increases the expression of CCND1 (a cell cycle regulatory protein, closely related to the progression of OA) by absorbing miR-142-5P in sponge, inhibits autophagy and apoptosis of OA chondrocytes, and promotes chondrocyte proliferation (Man et al., 2022). MELK, also a circRNA, is a novel pathogenic factor for OA. It sponges up miR-497-5P to regulate MYD88, a universal bridging protein involved in IL-1β-induced activation of NF-κB. MELK promotes OA chondrocyte apoptosis and inhibits autophagy using the miR-497-5P/MYD88/NF-κB signaling axis (Zhang et al., 2022). The levels of miR-140-5P, miR-149, and lncRNA SNHG are significantly lower in OA chondrocytes compared to those in the normal chondrocytes. Therefore, researchers came up with the idea of supplementing the levels of these three ncRNAs to explore their mechanisms in OA. Supplementation of miR-140-5P and miR-149 to overexpression downregulates the fucosyltransferase 1 (FUT1) and promotes the autophagy, accelerating the proliferation and inhibiting the apoptosis in human primary chondrocytes (Wang et al., 2018b). SNHG supplementation will regulate the synovial apoptosis inhibitor 1 (SYVN1) by targeting the absorption of miR-34a-5p and will promote cell proliferation and inhibit apoptosis. However, this process is realized by inhibiting autophagy rather than promoting it (Tian et al., 2020). Previous studies suggested that FBXO21, a member of the F-box, which presents a higher level in OA than in normal cartilage tissue, inhibits autophagy, increases apoptosis, and promotes cartilage degeneration. Jia et al. found that circR-MSR can inhibit autophagy in OA chondrocytes by targeting miR-761 to upregulate the FBXO21 level, resulting in OA (Jia et al., 2022). Furthermore, lncRNA HOTAIR sponges miR-130a-3p, leading to the upregulation of Bax and downregulation of Bcl-2, which inhibited autophagy, increasing apoptosis and decreasing cell viability (He and Jiang, 2020).

#### 5.2.4 ncRNAs regulate chondrocyte autophagy under different stresses

Due to the physiological structure, articular cartilage lacks capillaries, and oxygen levels in the joint are not high, which requires chondrocytes can maintain their physiological functions with an adaptive mechanism. Hypoxic conditions are conductive to the increase of ROS and oxidative stress and the overexpression of HIF-1α, leading to apoptosis of chondrocytes. Yang et al. found that targeting miR-411 negatively regulates HIF-1α, and inhibiting the expression of miR-411 elevates the levels of HIF-1α and autophagy-related Beclin-1, p62, ULK-1, and LC3. It has been confirmed that miR-411 promotes the chondrocyte autophagy by targeting HIF-1α, suggesting that miR-411 supplementation may be a new treatment method for OA (Yang et al., 2020a). Similarly, studies on the ncRNA/HIF-1α axis were also followed by Yang’s team, who found that p53 and HIF-1α compete in activating the transcription factors. A lncRNA ROR promoted the expression of HIF-1α, and p53 suppressed it. Based on the differential expression of autophagy and apoptosis-related factors, they suggested that ROR regulated apoptosis and autophagy in chondrocytes through HIF-1α and p53 (Yang et al., 2018).

Also, under hypoxia, Zhang et al. thought that miR-146a is a chondroprotective miR, confirming that HIF-1α and miR-146a promote the chondrocyte autophagy by inhibiting Bcl-2 (Zhang et al., 2015a). Besides, miR-146a has been reported with two inflammation-related targets (TRAF6 and IRAK1) and one target specific to chondrocytes (SAMD4). Chen et al. were surprised that in a hypoxic environment, miR-146a regulates Bcl-2 and autophagy via TRAF6 and IRAK1 instead of SMAD4 (Chen et al., 2017). Oxidative stress and inflammation are closely linked and easily accompanied (Biswas, 2016). TNF-α is an inflammatory factor, the TNF-α-induced cells exhibited a weaker ability but a more obvious apoptosis. miR-30b silencing can induce autophagy, reduce ECM degradation, and protect cells apoptosis under inflammatory response, as confirmed by Chen et al. through *in vitro* experiments. Besides, they found a direct interaction between miR-30b and the 3′UTR of Beclin-1 and ATG5 mRNA. In addition, they confirmed that miR-30b silencing protected the cells from apoptosis and inhibited ECM degradation through the upregulation of autophagy (Chen et al., 2016). miR-335-5p is associated with inflammatory response, with lower expression in OA chondrocytes. Expression of miR-335-5p can activate autophagy while reducing inflammatory mediators, thus significantly enhance the viability of chondrocytes (Zhong et al., 2019). In addition to oxidative stress and inflammatory stimuli, ERS can trigger autophagy (Lee et al., 2015), which is an important mechanism for cellular self-protection under ERS. Prolonged ERS, however, can cause accumulation of unfolded proteins that exceed the folding ability of the endoplasmic reticulum, ultimately leading to cellular dysfunction or death. Li et al. found that miR-375 could target the 3′-untranslated region of ATG2, block the expression of ATG2, inhibit autophagy, and promote ERS (Li et al., 2020a). In other words, inhibiting miR-375 increases the autophagy in response to ERS and alleviates the symptoms of OA. Table 2 lists the autophagy-related ncRNAs for OA treatment.

**TABLE 2:** ncRNAs for OA treatment by targeting autophagy.

ncRNA	Inhibition/activation of autophagy	Targets	Mechanism	References
miR-128	Inhibition	LC3 and ATG12	Promoting the chondrocyte apoptosis and inhibiting their proliferation	Sun et al. (2022)
miR-Let-7F	Inhibition	LC3 and Beclin-1	Promoting chondrocyte apoptosis	Feng et al. (2020)
miR-766-3p	Activation	LC3, Beclin-1, and p62	Promoting ECM formation and inhibiting chondrocyte apoptosis	Li et al. (2020d)
miR-206	Inhibition	PI3K/Akt/mTOR	Inhibiting chondrocyte apoptosis	Yu et al. (2019a)
miR-27a	Activation	PI3K/Akt/mTOR	Promoting chondrocyte apoptosis	Cai et al. (2019)
miR-20	Inhibition	PI3K/Akt/mTOR and ATG10	Inhibiting chondrocyte proliferation	He and Cheng (2018)
miR-155	Inhibition	ULK1, FOXO3, ATG14, ATG5, ATG3, GABARAPL1	—	D'Adamo et al. (2016)
miR-31-5p	Activation	SOX4/mTORC1	Inhibiting chondrocyte apoptosis	Xu et al. (2022)
miR-107	Activation	Akt/mTOR/NF-κB	Inhibiting chondrocyte apoptosis	Zhao et al. (2019)
miR-LOXL3	Inhibition	mTOR	Promoting chondrocyte apoptosis	Wei (2022)
miR-140-5p	Activation	LC3, ATG5, Beclin-1, and FUT1	Inhibiting chondrocyte apoptosis and promoting chondrocyte proliferation	Wang et al. (2018b)
miR-149	Activation	LC3, ATG5, Beclin-1, and FUT1	Inhibiting chondrocyte apoptosis and promoting chondrocyte proliferation	Wang et al. (2018b)
miR-411	Activation	LC3, Beclin-1, p62, and ULK1	Inhibiting chondrocyte apoptosis	Yang et al. (2020a)
miR-146a	Activation	Bcl-2	Protecting chondrocytes in hypoxic environment	Zhang et al. (2015a)
miR-30b	Inhibition	ATG5 and Beclin-1	Promoting ECM degradation and apoptosis	Chen et al. (2016)
miR-335-5p	Activation	ATG5, ATG7, and Beclin-1	Reducing inflammatory mediators and enhancing chondrocyte viability	Zhong et al. (2019)
miR-375	Inhibition	ATG2	Promoting oxidative stress	Li et al. (2020a)
lncR-CIR	Activation	LC3 and Beclin-1	Promoting the degradation of ECM	Wang et al. (2018a)
lncR-GAS5	Inhibition	miR-144/miR-21	Promoting chondrocyte apoptosis	Ji et al. (2021)
				Song et al. (2014b)
LncR- KLF3-AS1	Inhibition	PI3K/Akt/mTOR	Promoting chondrocyte apoptosis	Wen et al. (2022)
lncR-KCNQ1OT1	Activation	miR-128-3p/SIRT1	Inhibiting apoptosis	Sun et al. (2022)
lncR-POU3F3	Activation	miR-29-3p/FOXO3	Inhibiting chondrocyte apoptosis	Shi et al. (2022)
lncR-SNHG	Inhibition	miR-34a-5p	Inhibiting chondrocyte apoptosis and promoting chondrocyte proliferation	Tian et al. (2020)
lncR-HOTAIR	Inhibition	miR-130a-3p, LC3, and p62	Promoting chondrocyte apoptosis and weakening chondrocyte viability	He and Jiang (2020)
LncR-ROR	Activation	LC3 and Beclin-1	Inhibiting chondrocyte apoptosis	Yang et al. (2018)
circR-0037658	Inhibition	LC3, ATG5, Beclin-1, and p62	Promoting chondrocyte apoptosis and inflammatory response	Sui et al. (2021)
circR-RHOT1	Inhibition	miR-142-5p	Inhibiting chondrocyte apoptosis and promoting chondrocyte proliferation	Man et al. (2022)
circR-MELK	Inhibition	miR-497-5p/MYD88/NF-κB	Promoting chondrocyte apoptosis	Zhang et al. (2022)
circR-MSR	Inhibition	miR-761	Weaking chondrocyte viability	Jia et al. (2022)
Upregulate circR-7/downregulate miR-7	Inhibition	PI3K/Akt/mTOR	Promoting ECM degradation	Zhou et al. (2020b)

Abbreviations:ATG, autophagy-related protein; LC3, microtubule-associated protein 1 light chain 3; Beclin-1, and p62, the autophagy markers; ECM, extracellular matrix; mTOR, mammalian target of rapamycin; PI3K, phosphoinositide3 kinase; Akt, protein kinase B; ULK1, UNC-51-like kinase; FOXO3, Forkhead box O3; GABARAPL1, gamma-aminobutyric acid receptor-associated protein-like 1; mTORC, mammalian target of rapamycin complex; SOX4, SRY-related high-mobility-group box 4; NF-κB, nuclear factor-κB; FUT1, fucosyltransferase 1; SIRT1, sirtuins 1; MYD88, myeloid differentiation factor 88.

### 5.3 Autophagy-relate ncRNAs and RA

RA is characterized by inflammatory leukocytes white blood cells invading the fibrous vascular tissues from the blood, resulting in the erosive destruction of bone and cartilage (Lee and Weinblatt, 2001). Such erosive destruction is essentially a state of bone loss can be attributable to increased OC-mediated bone resorption and decreased OB-mediated bone formation due, causing an autoimmune response (Komatsu and Takayanagi, 2022). Meanwhile, RA is an autoimmune disorder that essentially relies on the interaction among the immune system, RA synovial fibroblasts (RASF), RA fibroblast-like synoviocytes (RA-FLS) and bone. To our knowledge, autophagy is closely related to some age-related diseases (Leidal et al., 2018), however, it also exerts a significant role in immune system diseases. In addition, autophagy is associated with the survival of patients with RASF, because it can activate various pro-inflammatory factors, and promote the secretion of RANKL (Wu and Adamopoulos, 2017), the latter of which further leads to OC activation and bone destruction (Takayanagi, 2021). Synovial tissue biopsy and autophagy-related molecular analysis showed higher autophagy-related factors and autophagy in synovial tissue of RA patients. It indicates that the autophagic pathway of RASF is significantly activated in the synovial tissue and autophagy is closely linked with the inflammatory response of RASF (Zhu et al., 2017). Also, autophagy also is closely related with bone destruction, and its relationship with OCs has been discussed before. In osteoclasts from RA patients, Foulquier et al. found high expression of ATG7 and Beclin-1 by immunofluorescence staining. It was also found that bone destruction was greatly decreased in ATG7 knockout mice compared with those in the wild-type mice. It was suggested that autophagy was also involved in the pathological process of OC bone destruction in RA patients (Foulquier et al., 2007).

There are few studies on autophagy-relate ncRNAs in RA, most of which focus on the apoptosis of RASF and RA-FLS. Autophagy displays a cytoprotective mechanism with a different purpose from apoptosis, and RASF has been shown to exhibit an anti-apoptosis ability (Firestein et al., 1995). In these cells, the autophagy pathways are upregulated and autophagy induction is associated with anti-apoptosis, thus no apoptosis of RASF may be associated with the upregulated autophagy (Shin et al., 2010). It was found that development of resistance to methotrexate (MTX) in RA-FLS may be associated with the upregulated autophagy; in fact, MTX-induced apoptosis was significantly increased when autophagy of RA-FLS was blocked (Xu et al., 2015). Furthermore, a similar study revealed a negative correlation between apoptosis and autophagy in RASF, which meant that the elevated Beclin-1 and LC3 expressions in RASF compared to osteoarthritic fibroblasts. It implied that an upregulation of autophagy may be associated with the dysregulation of miR-30a (Xu et al., 2013). These data suggest the important role of autophagy in RASF and RA-FLS anti-apoptosis and provide a new idea to manipulate autophagy in clinical treatment of RA. MTX, a commonly used drug for RA treatment clinically, induces autophagy and leads to apoptosis resistance. Most current drugs can only alleviate the symptoms rather than eliminate them, and most of them are based on the cytoprotein factors that target the activation or inhibition of RA. Actually, only 1%–2% of the human genome encode proteins. ncRNAs contribute to the diversity of gene expression (Lodde et al., 2020), can serve as drug targets, and can play a non-negligible role in RA. In addition, autophagy plays a key role in the RA treatment, so studies on autophagy-related ncRNAs in of the RA treatment emerge at the right moment. miR-218-5p was found to be highly expressed in RASF. Based on knockout of miR-218-5p, Chen et al. found increased expression of LC3-II and Beclin-1 and accelerated degradation of the autophagy substrate protein p62. However, silencing the transcription factor KLF9 regulates the oxidative stress, which reversed such phenomenon. It was confirmed that miR-218-5p could regulate proliferation, apoptosis, autophagy, and oxidative stress of RASF by directly targeting KLF9. In addition, they found that JAK/STAT3 may also be involved in the miR-218-5p/KLF9 axis to regulate the proliferation, apoptosis, autophagy, and oxidative stress of RASF (Chen et al., 2021). As described above, lncRNA ZFAS1 inhibits the osteogenic differentiation of BMSCs and sponges miR-2682-5p to regulate the cellular behavior of RA-FLS. Silencing ZFAS1 can decrease the LC3II, increase p62, and downregulate TNF-α and IL-6. These data confirmed that the expression of ZFAS1 promoted proliferation, autophagy, inflammatory response, and resists apoptosis in RA-FLS (Yang et al., 2020c). miR-146a is a chondroprotective ncRNA that plays a role in the bioactivity of RA-FLS. The expression of miR-146a weakens the autophagy and proliferation but enhances the apoptosis in RA-FLS through the PI3K/Akt/mTOR pathway. After treatment with Wenhua Juanbi Decoction, expression of miR-146a was inhibited, and the autophagy was regulated through the PI3K/Akt/mTOR pathway, which inhibited apoptosis (Zhou et al., 2022). This is different from the result that miR-218-5p inhibits autophagy and thus RASF apoptosis, which may be caused by different levels of autophagy. Inhibiting autophagy may increase the sensitivity of cells to apoptosis, and this balance between autophagy and apoptosis may be disrupted during the progression of RA. It suggests the precision of autophagy level may cause different outcomes when the ncRNAs and autophagy are combined for RA treatment. Table 3 lists the autophagy-related ncRNAs for RA treatment.

**TABLE 3:** ncRNAs for RA treatment by targeting autophagy.

ncRNA	Inhibition/activation of autophagy	Targets	Mechanism	References
miR-218-5p	Inhibition	LC3, Beclin-1, and p62	Inhibiting RASF apoptosis	Chen et al. (2021)
miR-146a	Inhibition	PI3K/Akt/mTOR	Promoting RA-FLS apoptosis and inhibiting its proliferation	Zhou et al. (2022)
LncR-ZFAS1	Activation	miR-2682-5p	Promoting proliferation and inflammatory response of RA-FLS	Yang et al. (2020c)

Abbreviations: RASF, rheumatoid arthritis synovial fibroblast; RA-FLS, RA fibroblast-like synoviocytes; LC3, microtubule-associated protein 1 light chain 3; Beclin-1, and p62, the autophagy markers; mTOR, mammalian target of rapamycin; PI3K, phosphoinositide3 kinase; Akt, protein kinase B.

### 5.4 Autophagy-related ncRNAs and other relatively rare bone metabolic diseases

OP, OA, and RA are some of the more common bone metabolism diseases in clinical practice, which affect the health of a large number of people. In addition, there are some bone metabolism diseases with relatively low incidence rates in the population, such as Osteolysis, Osteopetrosis, and Paget’s disease of bone.

Osteolysis is a severe complication following joint replacement surgery, and there is still inadequate understanding of its pathophysiology. However, it can be confirmed that its occurrence is related to the wear between the prosthesis and the surrounding bone. The wear particles generated in this process can lead to a series of pathological conditions, including inflammation, foreign body granuloma formation, and activation of osteoclasts, resulting in bone resorption and ultimately leading to the dissolution of bone tissue (Mbalaviele et al., 2017).

Autophagy has been demonstrated to be involved in osteolysis, and research indicates that wear particles can activate it in bone-related cells such as OBs, OCs, and macrophages (Wang et al., 2015). Autophagy can both promote and inhibit osteolysis, exhibiting a dual function (Li et al., 2018a; Wang et al., 2021), and there have been no studies of ncRNAs regulating autophagy to regulate osteolysis. Due to the dual nature of autophagy, it is essential to regulate autophagy to prevent osteolysis rationally, and it remains to be fully elucidated whether ncRNAs can be utilized to target autophagy so that they can play a positive role.

Osteopetrosis (OPT) is a rare hereditary bone disorder characterized by impaired function and differentiation of OCs, resulting in increased bone mass and abnormal bone structure (Pillai et al., 2022). Under normal circumstances, the function of OCs is to absorb and degrade bone tissue, which helps maintain normal bone remodeling and metabolic balance. However, in patients with OPT, genetic mutations disrupt the function and differentiation of OCs, increasing bone mass within the skeleton. Since OCs cannot absorb and degrade bone tissue normally, the bones of OPT patients become excessively dense (increased bone mass), with abnormal bone structure, making them brittle and prone to fractures. OPT can also lead to other symptoms, such as bone marrow failure, reduced production of red blood cells in the bone marrow, anemia, and neurological abnormalities (Del Fattore et al., 2008; Pillai et al., 2022). Bone marrow transplantation is currently the primary treatment method for OPT. The emergence of new mechanisms has brought about new therapeutic targets, with modulation of autophagy flux emerging as a potential target for treating OPT. Researchers have found autophagy defects in OPT mice, characterized by reduced expression of LC3-II and increased levels of p62 protein (Héraud et al., 2014). Additionally, studies have identified the PLEKHM1 gene, which plays a vital role in regulating intermediate autosomal osteopetrosis (an intermediate type of human osteopetrosis) and the endosomal/autophagy pathway in the human genome. It is involved in autophagosome formation, transport, and degradation through its interaction with LC3 and Rab7/HOPS proteins (Tabata et al., 2010; Héraud et al., 2014). Unfortunately, there is currently no research on the regulation of autophagy by ncRNAs for treating OPT.

Paget’s disease of bone (PDB) is a chronic focal bone disorder characterized by disrupted bone formation and increased bone resorption (Usategui-Martín et al., 2020). In PDB, the process of bone remodeling is abnormally activated, leading to abnormal reshaping of bone tissue. One observable feature is the presence of abnormally active OCs, specifically “overactive” giant OCs, which contribute to increased bone resorption (Reddy et al., 1999). While most individuals with PDB are asymptomatic, complications affecting the joints or nervous system can sometimes be observed. These complications may include pain, fractures, symptoms of nerve compression, and bone deformities (Alonso et al., 2017). Autophagy plays a vital role in regulating the bone resorption process within OCs. However, there is currently no definitive evidence to suggest a direct association between autophagy and PDB, and no studies have specifically investigated autophagy-related ncRNAs in this context. While mutations in p62 have been shown to inhibit autophagosome maturation, this finding only reinforces the hypothesis of autophagy involvement in PDB (Usategui-Martín et al., 2020). It is important to note that the pathogenesis of PDB involves multiple factors, including genetic, environmental, and cellular signaling pathway abnormalities (Gennari et al., 2022). Autophagy may be linked to some of these factors or pathways, but further research is needed to understand the specific relationships between them.

It can be observed that the research on these bone metabolic diseases mentioned above and even other rare bone metabolic diseases such as osteogenesis imperfecta, fracture nonunion, Craniometaphyseal Dysplasia, etc., have not yet been studied in depth, and there is a lack of in-depth research on the specific role and mechanisms of autophagy-related ncRNAs in their pathogenesis. However, we can draw on the achievements of current research on typical bone metabolic diseases (OP, OA, RA) to speculate on the potential roles of autophagy and ncRNAs in rare bone metabolic diseases, further reveal their pathophysiological mechanisms and provide guidance for the development of new treatment strategies.

### 5.5 Autophagy-related ncRNA: a significant target in the treatment of bone metabolic diseases with natural products

With the rise in medical and pharmaceutical-related illnesses and the shifting paradigms in health perspectives, the ingredients found in traditional herbal medicines and their derivatives are gradually gaining global acceptance owing to their minimal toxic side effects, affordability, and diverse structural properties. It is discernible from recent studies that these natural components can effectively modulate the expression of autophagy and ncRNAs, thereby playing a pivotal role in treating bone metabolic diseases. Researchers have demonstrated through *in vitro* and *in vivo* experiments that Zuogui Wan (a traditional Chinese medicine (TCM) in pilules) could upregulate the expressions of miRNA Let-7F and autophagy negative regulatory gene mTORC1, downregulate the expression of unc-51-like kinase 2 (ULK2) (a gene essential for initiating autophagy), and decrease the expressions of autophagy-related positive genes (Beclin-1, ATG12, ATG5, and LC3). It confirms that the Zuogui Wan can upregulate the Let-7F and inhibit the autophagy to accelerate the osteogenic differentiation of BMSCs (Shen et al., 2022). This shows that TCM is a “treasure trove” worth developing for OP treatment. Simiao San reversed the autophagy inhibition and apoptosis of chondrocytes caused by the low expression of Let-7E (Feng et al., 2020). Salvianolic acid B activates the chondrocyte autophagy and reduces the chondrocyte apoptosis through the KCNQ1OT1/miR-128-3p/SIRT1 signaling pathway (Sun et al., 2022). As a protective miR, miR-766-3p plays a crucial role in promoting and regulating the autophagy in addition to directly mediating the apoptosis-inducing factor mitochondrial associated Protein 1 (AIFM1) involved in the regulation of apoptosis. Studies have shown that miR-766-3p can promote autophagy and extracellular matrix synthesis, and alleviate the cell apoptosis. miR-766-3p can be upregulated by baicalin, an active ingredient in the Chinese medicine Scutellaria baicalensis, which in turn activates the autophagy and protects chondrocytes (Li et al., 2020d). It suggests that the natural product Scutellaria baicalensis exerts an unpredictable role in preventing and treating OA, which will be another reference solution using TCM. Wenhua Juanbi Decoction could promote the autophagy of RA-FLS and reduce apoptosis by inhibiting the expression of miR-146a.

From the studies mentioned above, it is evident that noteworthy are complex herbal concoctions like Zuogui Wan, Simiao San, and Wenhuajianbi Decoction, as well as active constituents derived from individual medicinal plants such as baicalin and salvianolic acid B, all of which modulate to varying degrees the autophagy and ncRNA expression in cells associated with bone metabolic diseases. Specifically, Zuogui Wan facilitates bone differentiation in BMSCs and hinders the progression of OP by upregulating miRNA Let-7F and autophagy negative regulatory gene mTORC1 expression, while downregulating the expression of autophagy-positive genes, thus inhibiting autophagy. Conversely, Simiao San, baicalin, and SAL B enhance cartilage cell survival and prevent the progression of OA by upregulating relevant ncRNAs, activating autophagy, and reversing cartilage cell apoptosis. Wenhuajianbi Decoction contributes to RA management by suppressing pertinent ncRNA expression, elevating autophagy levels, and RASF apoptosis.

Undoubtedly, natural products harbor immense potential in the treatment of bone metabolic diseases. However, their application is characterized by a degree of relativity and duality. The effective dosage, administration timing, and varying individual responses demand further exploration. Concurrently, the cytotoxicity and bioavailability of these herbal medicines remain under-researched, underscoring the critical need for meticulous dosage management in future clinical trials. Further targeted research is imperative to optimize clinical outcomes in the forthcoming period.

## 6 Conclusion

In a normal body, bone tissue constantly maintains the balance of bone remodeling and maintains bone homeostasis, which is inseparable from the normal function of BMSCs, OBs, osteocytes, OCs, and chondrocytes. Once such a balance is disrupted, bone homeostasis will be out of balance, resulting in a series of bone metabolic diseases, in which autophagy is a key treatment target. Many studies have recently reported on the regulation of autophagy by ncRNAs to treat bone metabolic diseases, including OP, OA and RA. Among them, miRNAs regulate target genes more directly than lncRNAs and circRNAs, which absorb and indirectly regulate target genes. Most studies on the regulation of bone metabolic diseases by autophagy-related ncRNAs focus on miRNAs which exert regulatory effects on autophagy through their specific targets and target-mediated downstream signaling pathways. Various miRNAs regulate different autophagy-related genes, and the same miRNA may also act at different targets in the same cell or tissue due to changeable stress conditions, which illustrates the sensitivity of miRNAs to environmental changes (Motta et al., 2013). Therefore, in the study of miRNAs regulating autophagy for bone metabolic diseases treatment, targeted studies should be developed according to different populations and environments. Moreover, for the same cells and the same signaling pathways, miRNAs may regulate the autophagy to different degrees and in multiple directions, either activating or inhibiting autophagy, thus affecting the biological activities of the cells. Besides, it is worth noting that the same biological process, such as the differentiation of OCs, can be regulated by multiple miRNAs whose functions either superimpose or compensate for each other. Unfortunately, it is not known exactly how they interact. Therefore, the possible side effects of different targets of miRNAs should be fully considered when the bone metabolic diseases are treated (Matsui and Corey, 2017). There are few studies on the regulation of autophagy by lncRNAs and circRNAs to affect bone metabolic disease. Most of these two ncRNAs affect their effects on targets by sponge adsorption of miRNAs, which makes the gene regulation in bone metabolism more complicated and may result in more unknown side effects. Perhaps, it can, in the future, concern on the direct regulation of target genes by lncRNAs and circRNAs.

As pathogenesis of bone metabolic diseases at the epigenetic level is becoming increasingly clearer, more studies have been triggered to address it. In this paper, three of the common bone metabolic diseases were reviewed, including OP, OA, and RA. Of course, more bone metabolic diseases should be included, such as osteolysis, Paget disease of bone, osteogenesis imperfecta, and delayed healing/non-healing fractures. However, there is a current paucity of research on autophagy-related ncRNAs for bone metabolic diseases, which will become a new frontier in non-coding genetic material bone metabolic diseases treatment by affecting autophagy.
